# Development and Characterization of Macerated and Non-Macerated Meads Made with Yeast with Probiotic Potential

**DOI:** 10.3390/foods15213802

**Published:** 2026-10-23

**Authors:** Esther Cañadilla-Valverde, Pilar Fernández-Pacheco, María Consuelo Díaz-Maroto, María Arévalo-Villena

**Affiliations:** 1 Department of Analytical Chemistry and Food Technology, Faculty of Chemical Sciences and Technologies, University of Castilla-La Mancha, Av. Camilo José Cela S/N, 13005 Ciudad Real, Spain; 2 Regional Institute for Applied Scientific Research (IRICA), University of Castilla-La Mancha, Av. Camilo José Cela 1, 13005 Ciudad Real, Spain

**Keywords:** honey fermentation, sequential inoculation, yeasts with probiotic potential, polyphenolic compounds, fruit maceration, volatile profile, sensory analysis

## Abstract

Mead is an alcoholic beverage made and consumed since ancient times and obtained by fermenting honey diluted in water. This fermented beverage has been gaining popularity in recent years, although it remains unfamiliar to many. The main objective of the present work was to develop meads (macerated and non-macerated) with a high polyphenolic content and a pleasant sensory profile by sequential inoculation of a non-*Saccharomyces* and a *Saccharomyces* strain with probiotic potential. For this purpose, the fermentative capacity of six separate yeasts, previously selected for their probiotic potential, was studied. Based on the physicochemical and sensory properties and the polyphenolic content of the meads obtained, the most suitable strains for sequential inoculation were UCLM-3 (*Saccharomyces cerevisiae*) and UCLM-1039 (*Lachancea thermotolerans*). With regard to the maceration of the final products, one mead was in contact with ginger and orange peel after fermentation, whereas another one received direct yeast inoculation without subsequent maceration. The first mead exhibited a higher polyphenol content, which was statistically different from that of the non-macerated beverage. Additionally, the volatile compounds present in both meads were identified and quantified. The macerated mead exhibited a higher terpene content, including compounds typically associated with ginger and orange peel. In the final stage of the research, a descriptive analysis and a hedonic test were carried out. The results showed that both beverages were well received by consumers.

## 1. Introduction

In recent years, there has been a notable increase in global interest in the consumption of fermented foods and beverages. This phenomenon can be attributed to the growing recognition of the biological functions of these products [1]. Fermented honey-based beverages are thought to be the oldest alcoholic beverages known to humankind. They were used in numerous Celtic, Anglo-Saxon, and Viking rituals and were even credited with healing powers, wisdom, and magical properties [2].

These beverages were made from the fermentation of honey diluted in water, containing between 8% and 18% ethanol (V/V). Although traditional mead consists simply of a fermented mixture of honey and water, other ingredients and additives (spices or fruit juices) are commonly added, offering numerous variations of the drink [2,3]. Therefore, the chemical characteristics of mead are closely related to and dependent on the type of honey used, the yeast strain responsible for fermentation, the nutrients added during the pre-fermentation stage, and the ingredients that may be used during the production of the product [3,4].

In this regard, the use of mixed cultures or co-cultures for the fermentation stage is a growing trend in beverage production. The procedure is based on the use of a *Saccharomyces* strain in combination with non-*Saccharomyces* yeasts. The implementation of this methodology aims to increase the quality and complexity of the products, making it easier to obtain various physical, chemical, and sensory characteristics [4]. In addition, the use of microorganisms that have beneficial characteristics for consumer health is of interest. Certain species of lactic acid bacteria are often highlighted as probiotic organisms, and there is growing interest in yeasts as probiotics. Probiotic yeasts not only improve food quality but can also increase the nutritional value of the product or food in which they are found through biofortification and the degradation of unwanted compounds or antinutrients [5] due to the probiotic and postbiotic compounds liberated.

Honey is also recognized as a functional food owing to its antioxidant and antimicrobial properties [6]. Its therapeutic effect is mainly due to the presence of several antioxidant and bioactive molecules, such as phenolic compounds, flavonoids, and phenolic acids [7,8]. The concentration of these phenolic compounds is closely related to the origin and type of honey and its concentration [3].

In addition, the maceration stage in the production of beverages allows the extraction of essential oils and/or flavors and aromas from the added ingredients, as the volatile compounds and other components present are solubilized in the alcohol. Therefore, macerating mead with ingredients such as spices, herbs, and fruits or their peels can increase the concentration of phenolic compounds in the beverage [9].

The reuse of by-products such as orange peel in the production of mead can offer numerous advantages, as it reduces waste, decreases environmental impact, and has the potential to increase bioactive compounds, generating added value in the final product [10]. Several antioxidant compounds have been found in citrus oils, mainly in the peel, in addition to phenolic acids and terpenes. The importance of these compounds is mainly due to their beneficial antioxidant, anti-inflammatory, antiproliferative, anti-allergic, antiviral, anticancer, neuroprotective, and antimicrobial properties [11]. As with orange peel, the nutritional and nutraceutical benefits associated with ginger are due to the presence of several bioactive compounds. These are classified into three classes: gingerols, volatile oils, and diarylheptanoids. Therefore, its use provides a wide range of beneficial compounds and offers distinctive organoleptic characteristics to the product [12].

Although the use of mixed *Saccharomyces*/non-*Saccharomyces* cultures has been widely investigated in wine and, to a lesser extent, in mead production, most studies have focused on fermentation performance, aroma development, and sensory quality. Comparatively little attention has been paid to the combined effect of probiotic-potential yeast co-cultures and post-fermentation maceration with plant-derived ingredients on the bioactive composition and sensory characteristics of mead. Furthermore, information regarding the influence of these strategies on polyphenol enrichment and volatile composition remains limited. Therefore, there is a need to evaluate whether the simultaneous application of sequential mixed-culture fermentation and maceration with valorized agro-industrial by-products can improve the functional and sensory attributes of mead.

Based on the bioactive properties of honey, orange peel, and ginger, we hypothesized that the combination of sequential mixed-culture fermentation and post-fermentation maceration would increase the polyphenol content and improve the volatile and sensory profile of mead. Therefore, the main objective of this study was to select a non-*Saccharomyces* and a *Saccharomyces* yeast strain, both with previously characterized probiotic potential, for sequential inoculation. In addition, macerated (with orange peel and ginger) and unmacerated meads were produced to evaluate and compare their polyphenolic content, volatile composition, and sensory characteristics.

## 2. Materials and Methods

### 2.1. Strains

A total of six yeast strains with probiotic potential were used (*Saccharomyces cerevisiae* UCLM-3, *S. cerevisiae* UCLM-137, *S. cerevisiae* UCLM-146, *Lachancea thermotolerans* UCLM-1039, *Hanseniaspora osmophila* UCLM-1056, and *Candida vini* UCLM-1063). These strains were isolated from different wineries and distilleries and were previously characterized and selected for their probiotic potential based on in vitro screening assays [13,14]. Specifically, the strains showed high tolerance to simulated gastrointestinal tract conditions and adequate kinetic parameters under these conditions. They also exhibited favorable adhesion-related properties, as assessed through autoaggregation, cell-surface hydrophobicity, and biofilm formation assays. Furthermore, their ability to adhere to Caco-2/TC7 cells was demonstrated. In addition, the safety of these strains was previously evaluated [15].

All strains were stored at −80 °C in the culture collection of the Yeast Biotechnology Laboratory (University of Castilla-La Mancha, Ciudad Real, Spain) and, prior to use, were revitalized in YPD (yeast extract, peptone, dextrose) broth (Condalab Pronadisa, Madrid, Spain) and incubated at 30 °C for 24 h. This medium was used in broth and agar, whose composition was 10 g/L yeast extract, 20 g/L glucose, 20 g/L peptone, and 20 g/L agar when used on plates.

### 2.2. Preparation and Inoculation of the Must

#### 2.2.1. Pure Culture Inoculation

The conditions for preparing the must were established based on various studies [16,17,18], with some modifications, as detailed below.

Holm oak honey (Muria^®^, Tarragona, Spain), also known as holm oak honeydew honey, was chosen as the base for the production of mead. This is artisanal holm oak honey from El Perelló (Tarragona, Spain).

For the production of must, the honey was diluted with water in a ratio of 1:3 until it reached approximately 23–25 °Brix, measured using a refractometer (Digital Pocket Refractometer, ATAGO CO., LTD., Tokyo, Japan). Tartaric acid was then added until a pH of 3.8 was reached, and the must was subsequently supplemented with diammonium phosphate (NH_4_)_2_HPO_4_ as a source of nitrogen (70 mg/L).

For the initial screening of the six yeast strains, three independent fermentations were performed for each strain. Each fermentation was considered an independent biological replicate. Then, the must was distributed into 18 previously sterilized flasks for the different strains under study and their respective triplicates, with each flask containing 100 mL of must.

Pasteurization was carried out at 60 °C for 30 min in a water bath. After that, the flasks were immediately cooled, and the different *Saccharomyces* and non-*Saccharomyces* strains were inoculated at a concentration of 2 × 10^6^ CFU/mL to test their capabilities individually.

Valves were placed on the flasks to facilitate gas (CO_2_) release and sampling while preventing oxygen from entering the system. Finally, each sample was incubated at 22 °C without agitation. The target fermentation endpoint was defined as an alcohol content of approximately 8–10% (V/V). Ethanol production was estimated from CO_2_ weight loss, and these calculations indicated that the target alcohol content was achieved after approximately 21 days of fermentation. Therefore, a fermentation period of 21 days was established for all experiments.

#### 2.2.2. Sequential Inoculation with the Selected Strains

Once the most adequate yeasts for sequential inoculation were chosen, the same procedure as described above was followed for the preparation of the must, except for the inoculation step, which in this case was sequential. First, 1 × 10^6^ CFU/mL of the selected non-*Saccharomyces* strain was inoculated, and the must was incubated at 22 °C. After 48 h, 1 × 10^6^ CFU/mL of the selected *Saccharomyces* strain was added. Three biological replicates were carried out.

#### 2.2.3. Fermentation Monitoring and Microbiological Analysis

Fermentation was monitored for each biological replicate by periodically measuring the weight (CO_2_ loss) every 24 h and the population count every 48 h. For the counts, the corresponding dilutions were made using a 0.9% saline solution and then seeded in drops on YPD plates in the case of must fermented with pure cultures. Plates of WL nutrient culture medium (a selective and differential medium) (Condalab Pronadisa, Madrid, Spain) were used to monitor the must fermented by sequential inoculation. This allows differentiation between *Saccharomyces* and non-*Saccharomyces* yeasts, as they exhibit different growth morphologies. These plates were incubated at 30 °C and counted after 48 h.

#### 2.2.4. Maceration of Mead and the Production of the Final Product

To obtain a homogeneous product before maceration, 7 L of must was fermented by sequential inoculation of the selected strains. After fermentation, the mead was subsequently divided into two treatments: non-macerated mead and macerated mead, and each treatment was divided into three different flasks (100 mL each one). The macerated samples were subjected to contact with orange peel and ginger (3 g/L of orange peel and 3 g/L of ginger for 24 h at 20 °C), whereas the non-macerated treatments were maintained without plant material.

Therefore, the maceration experiment started with the same mead, and each treatment (macerated and non-macerated) was designed to include three biological replicates.

### 2.3. Evaluation and Characterization of the Fermented Mead Samples

All the meads produced (initial screening with pure cultures, mead produced by sequential inoculation, and the final products obtained after sequential inoculation and maceration) were characterized as follows:

#### 2.3.1. Analysis of Physicochemical Parameters

A physicochemical analysis was performed on all samples using a WineScan™ FTIR analyzer (FOSS, Hillerød, Denmark) following the wine protocol to determine pH, ethanol content, and reducing sugars. The analyses were carried out using the instrument’s FTIR spectroscopic measurements and the corresponding calibration models for wine. Samples were analyzed according to the manufacturer’s instructions, and the values for ethanol (% V/V), pH (pH units), and reducing sugars (g/L) were automatically calculated by the instrument.

#### 2.3.2. Determination of Polyphenolic Compounds

The total polyphenols of each fermentation and maceration sample were determined according to the method described by Romano et al. [19] with some modifications.

First, 2.5 mL of Folin–Ciocalteu reagent previously diluted with distilled water (1:10) was added to 0.5 mL of the sample. The solution was left to stand for 3 min at room temperature. Next, 2 mL of sodium carbonate solution (10%) was added to each sample, and the samples were incubated for 15 min with stirring at room temperature. The absorbance was then measured at 765 nm using a UV-vis spectrophotometer.

Finally, the concentration of total phenolic compounds in the samples was determined using a gallic acid calibration curve. The calibration equation was y = 0.0011x − 0.0018, with a coefficient of determination R^2^ = 0.9942 and a linear range of 50–500 mg/L. A reagent blank, in which distilled water replaced the sample, was analyzed under the same conditions.

The results obtained were expressed in milligrams of gallic acid equivalents (GAE) per liter of sample.

It should be noted that the Folin–Ciocalteu assay is not specific for phenolic compounds, since other reducing substances present in the mead matrix, including reducing sugars, ascorbic acid and other compounds, may also contribute to the measured response. Therefore, the values obtained in this study are reported as estimated total phenolic content for comparison among samples and should not be interpreted as a direct measurement of individual phenolic compounds.

### 2.4. Determination of Volatile Compounds

Solid-phase microextraction followed by gas chromatography–mass spectrometry (SPE-GC-MS) was used to isolate, analyze and identify the volatile compounds in the final product samples, including both macerated and non-macerated mead.

#### 2.4.1. Solid Phase Extraction (SPE)

The active binding sites of the Supelclean Envi-Chrom P 05G/6 mL cartridges (Sigma-Aldrich, St. Louis, MO, USA) were activated following the manufacturer’s instructions (10 mL dichloromethane, 5 mL methanol, and 10 mL ethanol/water at 10%). After activation, the sample was prepared using a volume of 100 mL of mead, adding 40 μL of 4-nonanol (1 g/L in absolute ethanol) as an internal standard. The sample was introduced into the cartridges, and extraction was performed under vacuum conditions to facilitate its passage through the stationary phase. Subsequently, the hydrophilic compounds were eluted with Milli-Q water. Finally, the volatile compounds of interest were eluted with 10 mL of dichloromethane in tubes buried in ice to prevent their loss. The tubes were frozen to remove any traces of water eluted during extraction, and 24/48 h later, the organic phase was transferred to a heart flask by filtration with anhydrous sodium sulfate (desiccant) and glass wool. After that, the contents of the round-bottom flasks underwent a concentration process with a gentle stream of nitrogen to a final volume of approximately 200 μL. This volume was collected in vials fitted with conical inserts, which were duly labeled and stored under freezing conditions (−18 °C) until subsequent analysis by gas chromatography coupled with mass spectrometry (GC-MS).

#### 2.4.2. Analysis by GC-MS

In order to separate and identify all the volatile compounds previously extracted from the samples, a gas chromatograph coupled to an Agilent 6890GC/Agilent 5973 MD mass spectrometer (Agilent Technologies, Santa Clara, CA, USA) was used. An amount of 1 μL was injected in split mode (1/3) into an ultra-inert polar DB-WAX column (60 m × 0.25 mm i.d. × 0.25 μm stationary phase thickness) (Agilent Technologies, Santa Clara, CA, USA). The carrier gas was helium, maintaining a constant flow of 1.0 mL/min. The injector temperature was 250 °C, working in conjunction with a transfer line temperature of 280 °C. The oven temperature program was as follows: 70 °C for 5 min, ramped at 1 °C/min to 90 °C and held for 10 min, and increased at 2 °C/min to 210 °C and held for 40 min. The mass spectrometer operated by electron impact with an ionization energy of 70 eV, with an ion source temperature of 230 °C and SCAN mode (40–450 amu). Commercial standards (Sigma-Aldrich Chemie GmbH, Steinheim, Germany) together with the Kovats retention indices on the DB-WAX column, calculated using n-paraffins (C5-C26) as external references, were used to identify the volatile compounds. Quantification was based on calibration curves for each standard.

### 2.5. Sensory Analysis

#### 2.5.1. Meads Produced with Pure Cultures and Consensus Sensory Profile

A consensus sensory profile was carried out in accordance with the UNE-EN ISO 13299:2017 standard [20], in order to determine the strains that provided the best organoleptic properties for the selection of the mixed culture.

The panel was made up of eight trained tasters specializing in the evaluation of these kinds of products. All of them belonged to the staff of the Food Technology Department of the University of Castilla-La Mancha, Spain. The assessors had previous experience in sensory analysis and had undergone selection and training procedures in accordance with ISO 8586:2012 [21], including training in the identification, description, and evaluation of sensory attributes and the use of intensity scales.

In a preliminary session, the panelists selected the attributes that described the appearance, aroma, and flavor of the samples, as well as their intensities. Then, they proceeded to taste the meads fermented with each of the strains under study. The six samples were evaluated individually, using the symbol “+” (maximum 3) for the intensity of each attribute and the symbol “-” if the attribute was not present.

#### 2.5.2. Meads Produced by Sequential Inoculation and Maceration (Final Product)

A. Quantitative Descriptive Analysis (QDA)

A quantitative descriptive profile of the final product was made for the beverages produced in accordance with the UNE-EN ISO 13299:2017 standard [20]. Three tasting sessions (one for each replicate) were held, using the same trained panel but varying the sample codes in each session. Typical descriptors of appearance, smell, taste, and overall product quality were scored on a structured scale (0–9 points).

B. Hedonic Test with Consumers

In addition, a hedonic test was carried out with consumers in accordance with the UNE-EN ISO 11136:2017 standard [22] to evaluate consumer preference between macerated and non-macerated mead. In this case, a preference test was carried out according to the UNE-EN ISO 5495:2007 paired comparison test [23]. The consumer panel consisted of 64 participants, including 28 men and 36 women, with ages ranging from 20 to 60 years. Most participants reported consuming alcoholic beverages occasionally. All participants provided informed consent prior to inclusion.

The sensory evaluation was conducted over several sessions in a standard tasting room in accordance with ISO 8589:2007 [24], and the fermented beverages were presented using different identification codes.

### 2.6. Statistical Analysis

Statistical analysis of the results was performed using SPSS software (IBM SPSS Statistics 20) and Excel. Results were expressed as mean ± standard deviation (SD). All experiments were conducted in biological triplicate (*n* = 3). Depending on the experiment, the factors evaluated included yeast culture type and mead treatment (macerated and unmacerated mead). The response variables analyzed comprised microbial growth parameters, polyphenol content, volatile composition, and sensory attributes.

Prior to statistical analysis, normality was assessed using the Shapiro–Wilk test and by inspection of Q-Q plots, while homogeneity of variances was assessed using Levene’s test. No significant deviations from normality were detected, and homogeneity of variances was supported for the majority of compounds. Significant differences were studied at a 95% significance level using analysis of variance (ANOVA) and its corresponding post hoc test (Duncan’s test) when more than two groups were compared. Student’s *t*-test was used when only two groups were compared. To account for multiple comparisons, *p*-values were adjusted using the Benjamini–Hochberg procedure to control the false discovery rate (FDR), with an adjusted *p*-value < 0.05 considered statistically significant.

## 3. Results

### 3.1. Meads Produced with Pure Microbial Cultures

#### 3.1.1. Microbiological Analysis, Analysis of Physicochemical Parameters, and Determination of Polyphenolic Compounds

Counts of the initial and final yeast population, CO_2_ loss during fermentation, results of the physicochemical parameters (pH, reducing sugars, and alcohol content), and polyphenol contents after fermentation are shown in Table 1.

With regard to the final population, no significant differences were identified among the different strains. Both *Saccharomyces* and non-*Saccharomyces* strains demonstrated comparable growth patterns.

The pH values of the samples were found to be similar, ranging from 3.15 to 3.20. The only sample that demonstrated significant differences from the others was the mead fermented by *S. thermotolerans* UCLM-1039, with a pH value of 3.00, making it the most acidic sample. In fact, some studies, such as that by Fu et al. [25], indicate that *Lachancea thermotolerans* is often used in fermentation processes together with *Saccharomyces* in order to produce a rapid drop in pH.

Among the *Saccharomyces* yeasts, the corresponding samples with UCLM-137 and UCLM-146 showed the greatest total weight loss (CO_2_ generation), with values of 9.40 ± 0.30 g and 9.05 ± 0.15 g, respectively. However, *Saccharomyces* UCLM-3 was slightly slower, showing a total weight loss at 21 days of 7.45 ± 0.05 g. Therefore, the must inoculated with strains UCLM-137 and UCLM-146, which showed greater fermentative capacity, is expected to have a higher alcohol content and lower reducing sugar levels after 21 days.

With regard to the amount of reducing sugars and the alcohol content (related parameters), it was observed that almost all the samples showed significant differences between them. The mead fermented with *S. cerevisiae* UCLM-146 showed the highest alcohol percentage, 10.10% ± 0.03% (V/V), together with one of the lowest amounts of reducing sugars, 16.70 ± 0.14 g/L, corresponding to the predictions made previously, as well as to the results obtained from the fermentation with *S. cerevisiae* UCLM-137.

With respect to some non-*Saccharomyces* strains, Fu et al. [25] indicate that these yeasts have a low ethanol production yield due to the formation of a wide range of secondary metabolites, as could be the case with *C. vini* UCLM-1063, which showed a CO_2_ loss of only 6.40 ± 0.15 g. However, it is well known that the yeasts generally used for the fermentation process must belong to the *Saccharomyces* genus, because of their high fermentative activity and high tolerance, both to the osmotic pressure produced by sugars in the medium and to the high concentrations of ethanol [26]. Therefore, a higher alcohol content can be expected in samples fermented with yeasts of the *Saccharomyces* genus, as is the case with mead fermented with UCLM-146, showing significant differences compared with the other meads.

Sottil et al. [16] also studied the physicochemical parameters of meads fermented with pure *Saccharomyces* and non-*Saccharomyces* cultures. They observed a higher ethanol content, around 10.5% (V/V) for *S. cerevisiae* and 8.5% (V/V) for *Torulaespora delbrueckii*, with the latter having a higher residual sugar content, as is the case with the non-*Saccharomyces* strains tested in this study.

Strain UCLM-1063 had a low fermentation capacity, producing only 6.48% (V/V) of ethanol and leaving sugars unmetabolized (65.70 ± 0.08 g/L).

In addition, it was observed that *S. cerevisiae* UCLM-3 and *L. thermotolerans* UCLM-1039 showed similar fermentation processes (they did not show significant differences in reducing sugars), even though they belong to *Saccharomyces* and non-*Saccharomyces* yeasts, respectively.

Concerning the content of polyphenolic compounds, the samples corresponding to the non-*Saccharomyces* strains and *S. cerevisiae* UCLM-146 did not show significant differences between them, with values between 265.80 ± 3.26 mg/L and 277.22 ± 8.79 mg/L.

Nevertheless, the differences observed in total phenolic content among the meads should be interpreted with caution. Fermentation itself does not necessarily imply synthesis of phenolic compounds. Instead, differences in the values measured among strains may reflect strain-dependent effects on the release of phenolic compounds from bound forms, their adsorption to yeast cells, or their transformation and degradation during fermentation. In addition, the Folin–Ciocalteu assay is not specific for phenolic compounds, since other reducing substances present in the mead matrix, including sugars and other metabolites, may also contribute to the measured response. Nevertheless, the general trend showed that samples fermented with non-*Saccharomyces* yeasts had a higher phenolic concentration.

Furthermore, mead fermented with strain UCLM-137 showed a low concentration of polyphenolic compounds, being significantly different from the other meads, with a value of 227.43 ± 3.63 mg/L. Although this result cannot be conclusively explained, together with the other selection criteria, it contributed to the decision to exclude this strain from subsequent sequential inoculation experiments.

#### 3.1.2. Sensory Analysis and Consensus Sensory Profile

Table 2 shows the results obtained after performing the sensory analysis of the samples. Through consensus testing, the descriptors to be evaluated were first decided upon, and then their intensities were determined in each case, dividing the parameters into appearance, aroma, and flavor, in addition to considering the overall perception of the meads obtained.

With regard to the appearance descriptor, mead fermented with strain UCLM-1039 received the highest score (+++) for “brightness” and the lowest score (-) for “turbidity.” The samples fermented with UCLM-137 and UCLM-1063 also received the highest score for “brightness.” In terms of “color intensity,” the samples that stood out were those corresponding to the strains UCLM-146 and UCLM-1056.

The intensities assigned for aroma revealed that the only samples that did not receive the highest score for the descriptor “pleasant aroma” were samples fermented with UCLM-1056 and UCLM-1063. For the descriptor “honey,” the mead corresponding to UCLM-146 stood out above the rest. In addition, the sample fermented with UCLM-1039 was the only one in which floral notes were detected.

With respect to the flavor descriptors, the mead corresponding to the strain UCLM-1063 scored highest for both “sweet” and “honey,” which is consistent with the results obtained from the physicochemical analysis, as it had a high concentration of reducing sugars. The sample fermented with UCLM-137 received high intensities for “acidic” and “bitter,” thus becoming one of the worst-rated samples, along with the product obtained with UCLM-1063, in terms of overall perception, and these strains were therefore directly discarded for the selection of the starter culture.

Finally, regarding the overall perception of the samples, the meads obtained with UCLM-3 and UCLM-1039 were the best rated, both receiving the maximum score (+++).

### 3.2. Meads Produced by Sequential Inoculation

#### 3.2.1. Strains Selected

Several studies mention the beneficial role of fermentation carried out by non-*Saccharomyces* yeasts, as they can improve the aromatic profile, increasing the complexity and sensory quality of mead [27,28]. In addition, meads obtained by fermentation with *L. thermotolerans* have a high content of ethyl acetate, an ester associated with fruity and slightly floral aromas, and n-decanoic acid, a fatty acid that contributes to microbiological stability [27]. In fact, mead fermented with the UCLM-1039 strain was the only one that presented a floral aroma, which was positively evaluated when choosing it as one of the ideal candidates for sequential inoculation.

However, Cianí et al. [29] mention that some of these pure non-*Saccharomyces* yeast cultures can generate undesirable metabolites and fermentative characteristics, often leading to the exclusion of these types of strains in the coinoculation process. This could be the case for *C. vini* UCLM-1063, which received a poor rating in the sensory analysis and was therefore discarded. However, the study also mentions that, in mixed fermentations, some negative characteristics of non-*Saccharomyces* yeasts may not have been expressed or may have been modified by *S. cerevisiae* cultures.

The objective of the selection was to obtain a balanced mead with adequate fermentation performance and desirable sensory characteristics. Lower production of ethanol was not considered a disadvantage per se, since it is not an inconvenience in the development of a mead. Based on the results obtained in the study, UCLM-3 (*S. cerevisiae*) and UCLM-1039 (*L. thermotolerans*) were selected due to their overall performance considering fermentation behaviour, residual sugars, polyphenolic content and sensory characteristics. In particular, UCLM-1039 was considered of interest because of its sensory profile and its potential to contribute complementary characteristics to those provided by UCLM-3. Although the specific contribution of acid production by *L. thermotolerans* was not evaluated in the present study, strain selection was based on the combined assessment of technological and sensory criteria rather than on ethanol yield alone.

#### 3.2.2. Production of the Final Product

Once the strains of the starter culture had been selected, two types of mead were produced as final products: macerated and non-macerated mead.

To achieve this, 7 L of must was fermented by sequential inoculation of the selected strains. After fermentation, the mead was divided into two 3.5 L portions, corresponding to the macerated and non-macerated treatments, and each treatment was subsequently divided into three separate flasks. The maceration conditions were established based on preliminary tests, which concluded that the concentrations should be 3 g/L of orange peel and 3 g/L of ginger for 24 h at 20 °C.

##### Microbiological Analysis, Analysis of Physicochemical Parameters, and Determination of Polyphenolic Compounds

Table 3 shows the results obtained during this last stage, taking into account both the microbiological analysis and the physicochemical parameters, as well as the polyphenol content of the final products.

After fermentation, no significant differences were observed in the final yeast population, regardless of genus (*Saccharomyces* or non-*Saccharomyces*).

When producing the final mead, it was noted that the pH, reducing sugars, and alcohol content were significantly different from those obtained when the mead was produced by inoculating pure cultures.

When the must was inoculated sequentially with *L. thermotolerans* UCLM-1039 and *S. cerevisiae* UCLM-3, notable differences could be seen compared with the results obtained when these strains were used separately as pure cultures (Table 1). In this case, the mead had a lower alcohol content and a higher residual sugar content, as well as a more acidic pH than when the must was fermented only with *S. cerevisiae* UCLM-3 (31.50 ± 0.21 g/L of sugars, 8.69% ± 0.01% (V/V) ethanol, and a pH of 3.15 ± 0.03). This decrease in pH was due to the presence of *L. thermotolerans*.

In the work carried out by Gobbi et al. [30], the main analytical parameters of wines fermented with pure cultures of *S. cerevisiae* and *L. thermotolerans* were compared with mixed cultures of these strains under three different inoculation conditions. In all mixed fermentations, it was observed that ethanol production was significantly reduced compared with the *S. cerevisiae* control. This is consistent with the data obtained in the present study, since sequential inoculation of UCLM-1039 and UCLM-3 significantly reduced the alcohol content of the mead (0.8–1.0% [V/V]).

Another study conducted by Contreras et al. [31] observed that sequential inoculation of *Metschnikowia pulcherrima* and *S. cerevisiae* for wine production resulted in a product containing 1.6% (V/V) less ethanol than wine produced using pure *S. cerevisiae* as the starter culture. As in the previous case, this is consistent with our results.

Once the maceration process was concluded, the physicochemical parameters and polyphenol concentration of the samples were measured and compared.

No significant differences were observed in pH, as a value of *p* > 0.05 was obtained.

With regard to reducing sugars and alcohol content, significant differences (*p* ≤ 0.05) were observed between the meads. The slightly higher sugar content in the macerated mead may be related to the incorporation of small amounts of carbohydrates from the orange peel and ginger during the 24 h maceration period. Although a statistically significant difference in alcohol content was also observed, its magnitude was small (0.38% V/V). Therefore, this result should be interpreted with caution, as it is unlikely to represent a substantial technological or sensory difference between the beverages. Given the limited magnitude of the effect, the practical impact of maceration on ethanol content appears to be minimal under the conditions evaluated.

Finally, significant differences in phenolic content were observed between the final products (*p* < 0.05). It was noted that the macerated drink had a higher polyphenol content, with a value of 289.05 ± 5.10 mg/L. This increase may be associated with the release or extraction of reducing compounds from the orange peel and ginger during the 24 h maceration period, as both plant materials contain phenolic compounds and other reducing substances.

##### Determination of Volatile Compounds

The volatile composition of each of the beverages was isolated by solid-phase extraction (SPE) and then analyzed by gas chromatography coupled with mass spectrometry (GC-MS) to identify and quantify each of the compounds. The results obtained are shown in Table 4.

Twenty terpenes were identified, 13 of which were only present in the macerated mead samples. It should be noted that terpenes are naturally occurring volatile compounds widely found in plants and are particularly abundant in many aromatic botanicals. In the present study, the terpenes detected exclusively or at higher concentrations in the macerated meads are characteristic of the orange peel and ginger used for maceration. Their presence can therefore be mainly attributed to the extraction of these compounds from the added botanicals during maceration. Terpenes have also been reported in mead [32] and in monofloral honeys from citrus, lavender and eucalyptus [33], and their floral and fruity notes may contribute to the overall olfactory complexity of the macerated meads.

Linalool was identified as the predominant terpene in macerated mead. Other volatile compounds directly related to the presence of orange peel, such as α-pinene, γ-terpinene, α-terpineol, β-citronellol, and valencene, were found in smaller amounts [34,35,36]. The presence of zingiberene and citral is also noteworthy, resulting from the addition of ginger during maceration. Zingiberene and other compounds such as β-phellandrene and α-pinene are the main aromatic components of fresh ginger [37,38]. Many of the terpenes that were detected are known for their positive influence on the aroma of mead. Of those mentioned above, citral has a pleasant lemon aroma, and linalool has a floral and spicy aroma [32].

In addition, significant differences were observed in the total terpene content, with macerated mead having the highest concentration of these compounds. This increase is related to the high content of essential oils corresponding to the presence of orange peel and, to a lesser extent, ginger. In fact, orange peel is a known source of essential oils, which are usually composed of a complex combination of volatile compounds [39,40].

After the fermentation process, a complex mixture of flavor-active by-products is obtained, in addition to the production of ethanol and carbon dioxide by the yeast. Ethyl lactate and isoamyl acetate were the major compounds in this group in the two beverages produced [41]. In fact, the production of esters by yeasts during fermentation can have a significant effect on the fruity flavors of alcoholic beverages [42]. Compounds such as ethyl hexanoate and ethyl octanoate have apple and pear notes, while isoamyl acetate has a banana flavor. Ethyl esters are the most prevalent compounds in mead because ethanol is the most readily available substrate [32].

Concerning the alcohol group, the two main compounds within this group of volatiles for both samples were 2-methyl-1-propanol and 1-butanol, which did not show significant differences with respect to the type of beverage.

Various studies have established that alcohols constitute the predominant group of volatile compounds. In addition, 3-methyl-1-butanol was identified as the main compound in all the meads analyzed [27,43], with 2-methyl-1-propanol also standing out as one of the eight main volatile compounds in other studies [41].

The only compound that showed significant differences between the two beverages was 1-hexanol, which, according to the literature, is observed in higher concentrations in unpasteurized orange juice, so in our case it may be present in higher concentrations in macerated samples [35].

For the acid group, octanoic acid stood out, as in the study by Pereira et al. [44], in which six fatty acids were identified and quantified in all fermentations, with octanoic acid being the main compound.

Chitarrini et al. [32] also studied mead that was produced using honeydew honey. They found that, after fermentation, the mead was richer in acids, such as hexanoic acid, octanoic acid, and decanoic acid, and ethyl esters, such as ethyl octanoate, isoamyl hexanoate, ethyl nonanoate, ethyl decanoate, and ethyl dodecanoate.

Three furanic compounds, furfural, 2-acetylfuran, and coumaran, were identified in both types of meads. These compounds are naturally present in honey, with their concentrations increasing notably under elevated storage temperature [45], and they could exert an inhibitory effect on yeasts at concentrations much higher than those found in our meads [46].

Finally, with regard to benzene compounds, it is important to note that 2-phenylethanol was significantly higher than the other compounds. This compound also stood out in other studies related to the composition of volatile compounds in mead [41]. In fact, 2-phenylethanol generally contributes positively to the aroma of fermented beverages, characterized by a pleasant rose aroma [42].

Furthermore, 2-phenylethanol, together with ethanol and other alcohols, influences the taste of alcoholic beverages, creating a warm sensation in the mouth [47].

Two benzene compounds, eugenol and methyl eugenol, with a clove-like aroma, were detected exclusively in the macerated meads. Their presence is attributed to the extraction of phenylpropanoids from the botanical ingredients introduced during the maceration process, mainly from ginger [48].

##### Sensory Analysis and Quantitative Descriptive Profile

A descriptive sensory analysis was carried out by the same panel with the main objective of determining the sensory profile of the products produced, thus determining whether there were significant differences between them.

The experts evaluated the different attributes based on the appearance of the samples and the olfactory and taste sensations, assessing the overall quality at the end of each session.

**Figure 1:**
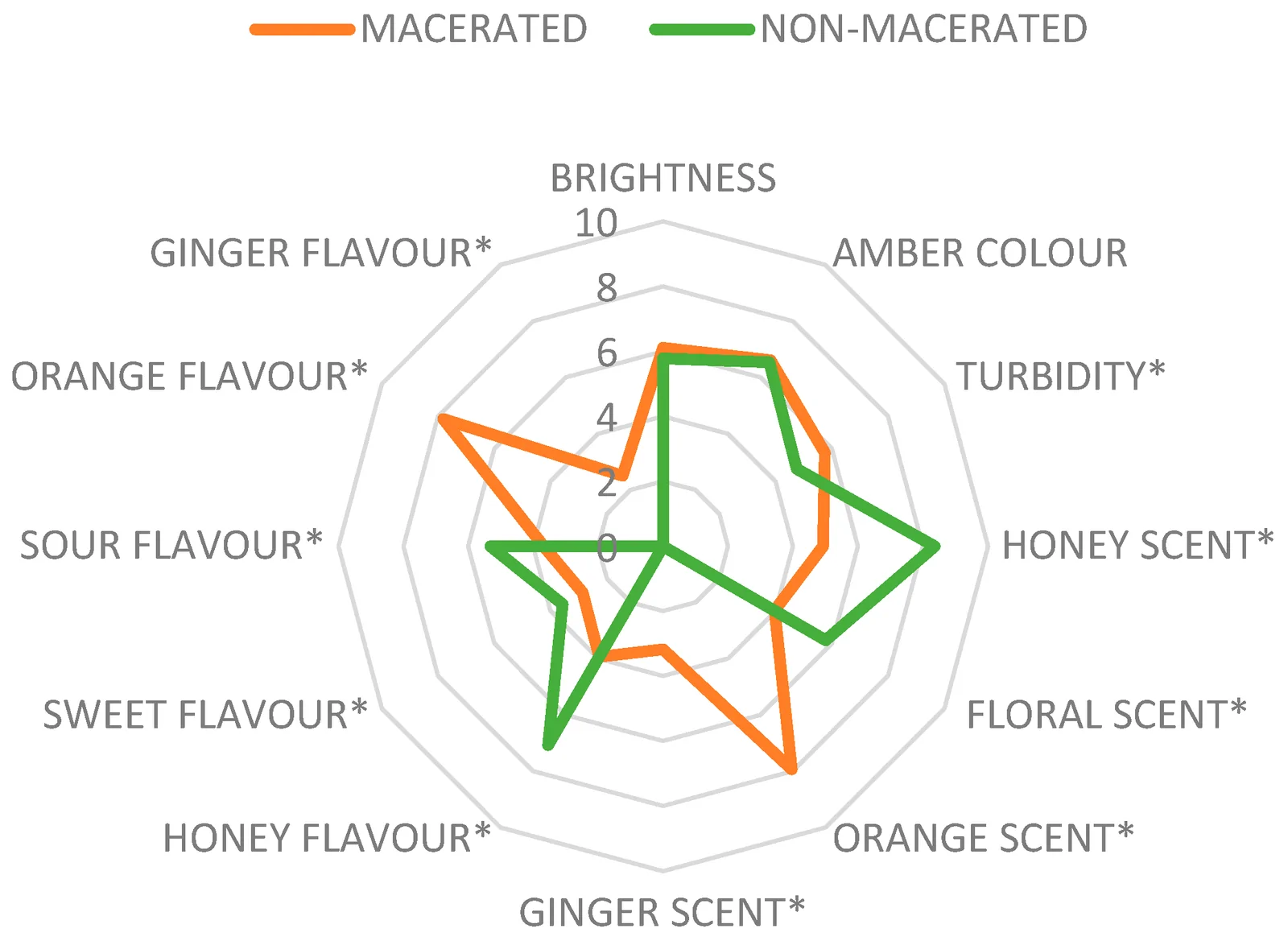
Results obtained in the descriptive profile of the processed products: appearance, olfactory sensation, and taste sensation. The asterisk (*) indicates significant differences according to Student’s *t*-test (*p* < 0.05).

Figure 1 shows the results obtained after two tasting sessions.

A 9-point discrete unipolar scale was used. Each attribute was studied separately using Student’s *t*-test to determine whether there were significant differences between the two products.

In terms of appearance, both products received similar scores, with no significant differences in the attributes “brightness” (*p* > 0.05) and “amber color.” In both cases, the scores given by the tasters ranged from 5 to 7 points. However, significant differences in “turbidity” were observed (*p* < 0.05), with the macerated mead receiving a higher score (5–6 points) for this attribute, as the samples were not centrifuged after maceration, leaving some sediment.

With regard to the olfactory sensation, each attribute was mainly related to one of the two meads. The non-macerated mead was associated with the attributes “honey” and “floral,” while the macerated product received the descriptor “orange” with a score of 7–8 points, and the descriptor “ginger” obtained scores ranging from 1 to 2 points. According to the statistical analysis, all attributes showed differences between the two products.

The results on taste sensation assigned the attributes “honey,” “sweet,” and “acidic” to the unmacerated mead. Meanwhile, macerated mead was again associated with “orange” and “ginger,” showing very similar scores to those obtained for olfactory sensation (7–8 points and 2–3 points, respectively, for each attribute). As in the previous case, all attributes showed significant differences between the two products.

In terms of the overall score, the macerated product received scores between 8 and 9 points, and the non-macerated product between 6 and 8 points. The macerated mead was the highest-rated product.

##### Hedonic Test with Consumers

The data collected in the hedonic test were analyzed using the tables provided in Annex A of the UNE-EN ISO 5495:2007 standard (paired comparison test) [23] as a reference.

For *n* = 64 tasters and a significance level of α = 0.05, a minimum of 40 “correct” responses is required to conclude that there is an appreciable difference between the meads produced.

After analyzing the data, significant differences between the samples were observed for appearance and smell. The non-macerated mead was preferred in terms of appearance, whereas the macerated mead was preferred in terms of smell. However, there were no differences in terms of “taste.” Thirty participants selected the macerated product, whereas 34 selected the non-macerated product. Similarly, the overall assessment of preferences did not reveal a significant preference for either of the two meads, although it showed that this choice aligned with the choices made based on the taste of the samples.

## 4. Conclusions

The screening of yeast strains with previously characterized probiotic potential enabled the selection of *L. thermotolerans* UCLM-1039 and *S. cerevisiae* UCLM-3 as a suitable combination for sequential inoculation in mead production, which was supported by the complementary technological and sensory characteristics studied.

Sequential inoculation of the selected strains successfully produced mead with a lower alcohol content and higher residual sugar content than those obtained with the corresponding pure cultures. Beyond the fermentation strategy itself, post-fermentation maceration with orange peel and ginger proved to be an effective approach for further modulating the flavor characteristics of the beverage. The macerated mead showed a higher estimated total phenolic content and a markedly different volatile profile, characterized by a greater concentration and diversity of terpenes associated with the plant ingredients used. These compositional changes resulted in clearly differentiated sensory profiles, with orange and ginger notes characterizing the macerated mead, and honey, sweet, and floral attributes being more closely associated with the non-macerated product. Consumer evaluation further showed that the two products generated different preferences depending on the sensory attribute considered, although no significant overall preference was observed.

The combination of yeast selection, previously characterized probiotic potential, sequential inoculation, and post-fermentation maceration with orange peel and ginger offers a promising approach for diversifying mead production and developing fermented beverages with differentiated composition and sensory profiles.

These findings should nevertheless be considered preliminary, as the study included a limited number of biological replicates and the monitoring during storage are necessary to confirm the reproducibility and stability of the developed beverages. Finally, although the selected strains had previously demonstrated probiotic potential in vitro, this property was not evaluated in the final meads, and future studies should determine whether their previously characterized probiotic-related properties are maintained in the final product and throughout storage.

## Figures and Tables

**Table 1:** Musts fermented with pure cultures. Initial and final yeast population counts. Physicochemical parameters after 21 days of fermentation. Determination of polyphenolic compounds in the samples (*n* = 3). The superscripts (a–e) indicate significant differences between the values in the columns according to Duncan’s test (*p* < 0.05) for each of the parameters.

	Inoculated Strain					
	*Saccharomyces*			Non-*Saccharomyces*		
Analysis/Parameters	UCLM-3	UCLM-137	UCLM-146	UCLM-1039	UCLM-1056	UCLM-1063
**Population count**						
**Initial population (log CFU/mL)**	6.03 ± 0.00					
**Final population (log CFU/mL)**	8.04 ± 0.41	7.97 ± 0.54	7.86 ± 0.78	7.67 ± 0.92	7.85 ± 0.58	7.72 ± 0.75
**Physical–chemical parameters**						
**pH**	3.15 ± 0.03 ^a^	3.20 ± 0.04 ^a^	3.18 ± 0.04 ^a^	3.00 ± 0.02 ^b^	3.19 ± 0.02 ^a^	3.17 ± 0.03 ^a^
**Weight loss (g)**	7.45 ± 0.05 ^c^	9.40 ± 0.30 ^a^	9.05 ± 0.15 ^a^	8.3 ± 0.21 ^b^	8.20 ± 0.20 ^b^	6.40 ± 0.10 ^d^
**Reducing sugars (g/L)**	31.50 ± 0.21 ^b^	15.80 ± 0.07 ^e^	16.70 ± 0.14 ^d^	31.50 ± 0.23 ^b^	24.50 ± 0.15 ^c^	65.70 ± 0.08 ^a^
**Alcohol content % (V/V)**	8.69 ± 0.01 ^d^	9.79 ± 0.03 ^b^	10.10 ± 0.03 ^a^	8.95 ± 0.04 ^c^	9.81 ± 0.01 ^b^	6.48 ± 0.02 ^e^
**Determination of polyphenolic compounds**						
**Concentration (mg/L)**	256.72 ± 3.06 ^b^	227.43 ± 3.63 ^c^	269.17 ± 2.74 ^a^	265.80 ± 3.26 ^a,b^	277.22 ± 8.79 ^a^	272.36 ± 2.02 ^a^

**Table 2:** Sensory analysis: consensus tests of the samples produced. The intensity of each attribute was evaluated using the following scale: (-), absence of the attribute; (+), low intensity; (++), moderate intensity; and (+++), high intensity (maximum score).

		Inoculated Strain					
Descriptors		UCLM-3	UCLM-137	UCLM-146	UCLM-1039	UCLM-1056	UCLM-1063
**Appearance**	Brightness	+	+++	++	+++	++	+++
	Colour intensity	++	+	+++	+	+++	++
	Turbidity	+	+	++	-	++	+
**Aroma**	Honey	+	++	+++	++	+	-
	Winey	++	++	+	+	+	+
	Pleasant aroma	+++	+++	+++	+++	++	+
	Floral	-	-	-	++	-	-
**Flavour**	Sweet	++	+	+	+	++	+++
	Acid	+	+++	++	++	++	-
	Bitter	-	++	+	-	++	-
	Honey	+++	+	++	++	+	+++
**Balanced/** **Overall perception**		+++	+	++	+++	++	+

**Table 3:** Physicochemical parameters after 21 days of fermentation (*n* = 2). Initial and final yeast population counts. Weight loss (CO_2_ generated). Determination of polyphenolic compounds in the final product without maceration and with maceration. The asterisk (*) indicates significant differences between samples according to Student’s *t*-test (*p* < 0.05).

		Sample Type	
Analysis/Parameters		MEAD	
**Population Count**			
**Initial population (log CFU/mL)**	**UCLM-3**	6.00 ± 0.00	
	**UCLM-1039**	6.00 ± 0.00	
**Final population (log CFU/mL)**	**UCLM-3**	7.88 ± 0.74	
	**UCLM-1039**	8.65 ± 0.48	
		**Weight loss (g/L)**	
**7 L fermented mead**			148.53 ± 5.60 g
		**NON-MACERATED MEAD**	**MACERATED MEAD**
**Physical–chemical parameters**			
**pH**		2.95 ± 0.02	2.97 ± 0.01
Reducing sugars (g/L) *		59.83 ± 0.07	60.6 ± 0.03
Alcohol content (% V/V) *		7.71 ± 0.02	7.33 ± 0.02
**Determination of polyphenolic compounds**			
Concentration (mg/L) *		271.17 ± 4.16	289.05 ± 5.10

**Table 4:** Volatile components (µg/L) in non-macerated mead (MS) and macerated mead (MM) (*n* = 3). The asterisk (*) indicates statistically significant differences according to Student’s *t*-test (*p* < 0.05).

	RI Calculated	MS	MM
		Mean ± SD	Mean ± SD
α-pinene *	1024	Nd	14.40 ± 1.00
δ-3-carene *	1150	Nd	5.20 ± 0.50
β-myrcene *	1160	Nd	57.02 ± 1.04
γ-terpinene *	1243	Nd	2.61 ± 0.17
*cis*-linalool oxide	1435	24.28 ± 1.86	24.72 ± 2.12
*trans*-linalool oxide	1462	7.08 ± 1.69	8.84 ± 0.72
linalool *	1544	5.49 ± 0.07	158.74 ± 6.75
carophyllene *	1574	Nd	4.47 ± 0.22
4-terpineol *	1583	Nd	32.78 ± 1.13
α-terpineol *	1677	Nd	17.99 ± 1.90
endo-borneol *	1679	Nd	7.35 ± 0.25
valencene *	1689	Nd	15.68 ± 1.44
zingiberene *	1696	Nd	9.99 ± 0.86
citral *	1707	Nd	9.89 ± 0.43
β-citronellol *	1830	Nd	7.84 ± 1.08
2,6-dimethyl-3,7-octadiene-2,6-diol	1942	29.59 ± 4.64	27.54 ± 5.38
thymol	2172	7.05 ± 0.76	6.76 ± 0.38
hydroxylinalool	2306	57.67 ± 2.45	57.37 ± 4.22
geranic acid *	2327	Nd	25.45 ± 1.04
farnesol	2350	3.74 ± 0.12	4.81 ± 0.66
TOTAL TERPENES *		134.89 ± 4.06	499.45 ± 17.71
ethyl butanoate	1029	19.99 ± 5.63	28.41 ± 3.54
isoamyl acetate	1121	233.35 ± 12.52	227.39 ± 13.10
ethyl hexanoate	1231	32.5 ± 6.40	26.67 ± 1.64
ethyl lactate	1341	1830.50 ± 240.15	1628.69 ± 52.44
ethyl octanoate	1427	68.07 ± 5.28	69.89 ± 12.08
ethyl nonanoate	1532	8.93 ± 0.53	9.33 ± 0.77
ethyl dodecanoate	1840	16.58 ± 2.05	24.09 ± 2.69
ethyl tetradecanoate	2044	23.01 ± 1.41	22.88 ± 1.85
ethyl hexadecanoate	2251	90.66 ± 10.43	103.00 ± 9.85
ethyl octadecanoate	2456	156.52 ± 20.86	141.04 ± 17.77
ethyl oleate	2475	120 ± 16.54	120.27 ± 14.56
diethyl succinate *	1656	24.36 ± 3.37	40.88 ± 1.20
TOTAL ESTERS		2623.97 ± 236.38	2442.54 ± 97.31
2-methyl-1-propanol	1084	269.63 ± 74.64	229.14 ± 32.06
1-butanol	1139	573.74 ± 110.32	485.59 ± 36.90
1-pentanol	1247	54.68 ± 1.29	52.21 ± 2.60
3-methyl-1-pentanol	1326	60.17 ± 1.32	56.91 ± 2.24
1-hexanol *	1351	8.17 ± 1.48	20.98 ± 1.11
3-ethoxy-1-propanol	1375	38.71 ± 11.62	24.47 ± 0.38
3-methylthiopropanol	1695	46.85 ± 6.36	44.03 ± 1.51
TOTAL ALCOHOLS		1051.96 ± 198.96	913.32 ± 65.30
acetic acid	1438	50.83 ± 7.57	56.24 ± 14.28
2-methylpropanoic acid	1556	66.91 ± 19.64	48.52 ± 10.02
hexanoic acid	1830	195.05 ± 6.86	186.26 ± 9.08
octanoic acid	2049	399.79 ± 25.04	377.77 ± 23.01
nonanoic acid	2154	23.58 ± 4.83	27.53 ± 2.21
decanoic acid	2263	85.98 ± 7.47	96.32 ± 0.66
TOTAL ACIDS		822.14 ± 60.42	792.64 ± 58.45
furfural	1446	10.47 ± 1.17	9.51 ± 0.21
2-acetylfuran	1490	6.75 ± 0.59	7.47 ± 0.34
coumaran	2377	84.44 ± 13.00	57.07 ± 8.81
TOTAL FURANIC COMPOUNDS		101.66 ± 13.01	74.05 ± 9.25
benzaldehyde	1507	7.07 ± 1.01	11.19 ± 2.41
2-phenylethanol	1901	26,442.22 ± 3272.24	25,636.00 ± 623.87
benzothiazole	1938	24.01 ± 3.84	19.72 ± 5.94
methyl eugenol *	2003	Nd	22.16 ± 1.79
eugenol *	2151	Nd	20.73 ± 2.33
4-vinylguaiacol *	2178	78.13 ± 5.59	181.24 ± 18.67
isoeugenol	2333	14.34 ± 3.51	9.32 ± 2.57
benzoic acid	2411	256.85 ± 24.58	237.98 ± 20.69
benzeneacetic acid *	2538	1141.04 ± 88.46	725.62 ± 101.02
TOTAL BENZENE COMPOUNDS		27,963.66 ± 3344.86	26,852.77 ± 769.33

RI: retention indices calculated (DB-Wax). Nd: not detected.

## Data Availability

The original contributions presented in this study are included in the article. Further inquiries can be directed to the corresponding author.
